# Implementation of telemedicine for knee osteoarthritis: study protocol for a randomized controlled trial

**DOI:** 10.1186/s13063-018-2625-4

**Published:** 2018-04-17

**Authors:** Zhengping Huang, Xia Pan, Weiming Deng, Zhixiang Huang, Yukai Huang, Xuechan Huang, Zhaohua Zhu, Weiyu Han, Shaoling Zheng, Xin Guo, Changhai Ding, Tianwang Li

**Affiliations:** 1 Department of Rheumatology and Immunology, Guangdong Second Provincial General Hospital, Guangzhou, China; 2 Clinical Research Centre, Zhujiang Hospital, Southern Medical University, Guangzhou, Guangdong China; 3 Menzies Institute for Medical Research, University of Tasmania, Hobart, TAS Australia

**Keywords:** Osteoarthritis, Telemedicine, GOH, Disease management, Randomized clinical trial

## Abstract

**Background:**

Osteoarthritis (OA) is the most prevalent chronic joint disease, characterized by joint structural deterioration, pain and loss of function among the elders. It is also associated with several extra-articular symptoms (fatigue, sleep disorders, anxiety and depression) and a reduction of life quality. Studies have revealed that patients with OA benefitted from enhanced management via telemedicine. Guangdong Online Hospital (GOH) is the first officially recognized web-based hospital that provides telemedicine service in China. However, the effective implementation of GOH telemedicine (GOHT) to enhance management for patients with OA remains unknown.

**Methods/design:**

An assessor-blinded, parallel randomized controlled trial will be performed to study the feasibility and effectiveness of GOHT in the enhanced management of OA. Forty participants with knee OA will be recruited for a 6-month study. Patients meeting the inclusion criteria will be randomly allocated to receive conventional therapy (CT) or conventional therapy plus a brief GOH-based intervention (CT-GOHT). The primary outcome is the feasibility of a full-scale randomized controlled trial. The secondary outcomes include the self-reported total score of the Western Ontario and McMaster Universities Osteoarthritis Index (WOMAC), the Multidimensional Fatigue Inventory (MFI), the Pittsburgh Sleep Quality Index (PSQI) and the Hospital Anxiety and Depression Scale (HADS). Assessments will be performed at baseline, 2 weeks, 3 months and 6 months later after the initiation of the study.

**Discussion:**

This trial is intended to test the application of GOHT in the chronic management in knee OA. The hypothesis is that OA patients may receive disease management via this network platform conveniently and effectively, especially those in the remote areas of our country. GOHT telemedicine would be an attractive alternative to traditional methods for disease management in knee OA. The results could provide preliminary experiences and guidance for an upcoming full-scale randomized controlled trial (RCT) in disease management via telemedicine.

**Trial registration:**

ChiCTR: ChiCTR1800014465. Registered on 16 January 2018.

**Electronic supplementary material:**

The online version of this article (10.1186/s13063-018-2625-4) contains supplementary material, which is available to authorized users.

## Background

Osteoarthritis (OA) is a degenerative joint disease that involves the whole joint structure, including synovium, cartilage, menisci, ligaments, periarticular muscle, capsule and subchondral bone [1]. Globally, the age-adjusted prevalence of knee OA is 3.8% and of hip OA is 0.85% [2]. In China, the prevalence of symptomatic knee OA among people aged 45 years or older is 10.3% in women and 5.7% in men [3]. OA causes joint pain, functional impairment and disability [4]. Moreover, OA patients suffer from a series of extra-articular symptoms. It is reported that poor sleep is highly prevalent in OA patients and approximately 40% of OA patients have substantial fatigue which is related to pain, sleep disturbance and depressed mood [5, 6]. Besides, a considerable number of OA patients experience clinically significant depression and anxiety [7, 8]. OA has become a very serious medical problem that significantly decreases the quality of patients’ life and causes large social and economic burden [9].

As a prevalent chronic disease, OA patients are in need of long-term disease management to improve their quality of life and to prevent disability, and this demand is expected to increase greatly in the coming years with the rising incidence. As an emerging concept in health care, telemedicine, provides ways to remotely deliver consultations, treatments and monitoring disease activities [10]. Telemedicine has been shown positive results in feasibility, effectiveness and patient satisfaction in the use of chronic disease management [11, 12]. Evidence has shown that Internet-based interventions significantly improve health status and meet with high acceptance and high user satisfaction in patients with OA [13].

As an institution of Guangdong Second Provincial General Hospital, Guangdong Online Hospital (GOH) is China’s first officially recognized online hospital. Clinicians of almost all medical departments are available at this online platform (http://www.youdeyi.com/). Patients are able to receive online health services such as consultations, disease education and preliminary treatment suggestions through the telemedicine platforms with audio and video settings in the communities around the province. Our previous study has demonstrated that GOH provided a convenient platform for ankylosing spondylitis patients to receive effective disease management, especially those in the remote areas of the country [14]. The positive results encourage us to explore the role of GOHT telemedicine in the management of OA.

Therefore, we design this trial to examine the feasibility and effectiveness of GOH for knee OA management in the context of a routine clinical care. We hypothesize that GOHT can serve as a convenient telemedicine platform providing disease-enhanced management for OA patients.

## Methods/design

### Trial design

**Fig. 1:**
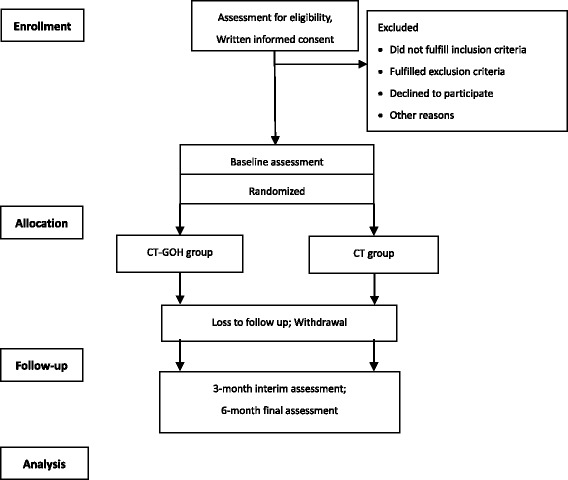
Flow chart of trial participation

**Fig. 2:**
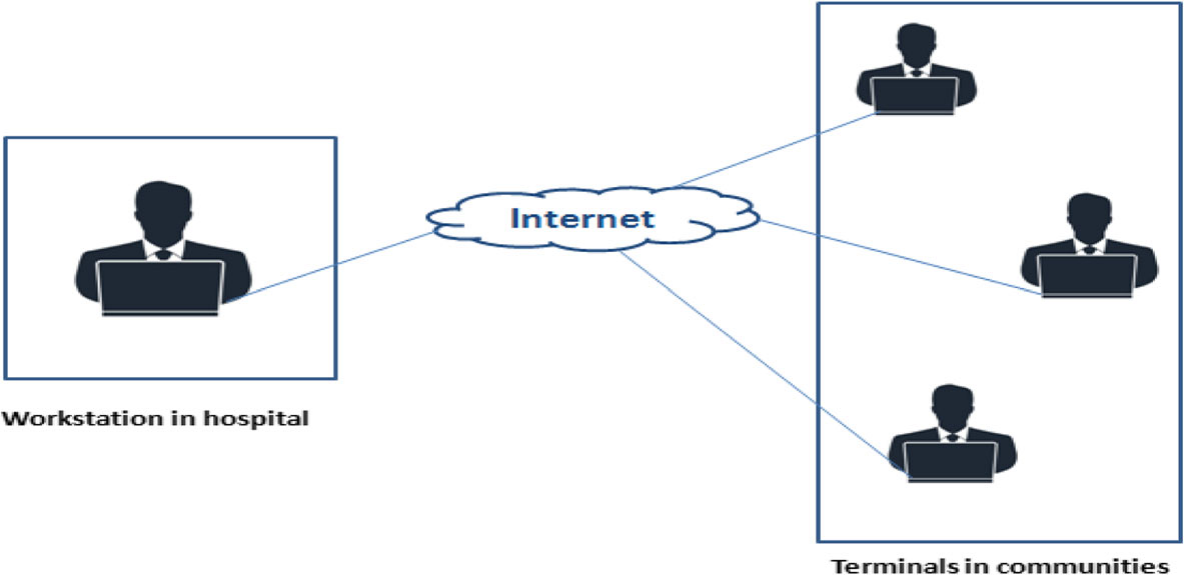
The operating mode of Guangdong Online Hospital (GOH)

This study is a prospective, parallel randomized controlled trial (RCT) conforming to Standard Protocol Items: Recommendations for Interventional Trials (SPIRIT) guidelines (Additional file 1) [15]. The flow chart of the participants is illustrated in Fig. 1. This study will be carried out in our hospital (Fig. 2). The total duration of the study is expected to take approximately 12 months depending on the process of enrollment and follow-up visits. Both groups will be followed up for 6 months.

### Participants

#### Inclusion criteria

Participants will be qualified for inclusion when they meet the following criteria: meeting the 1986 American College of Rheumatology (ACR) classification criteria for knee OA [16]; knee OA with a pain score of at least 20 mm on a 100-mm Visual Analogue Scale (VAS) for at least 6 months [17]; Kellgren and Lawrence grades 0–3 [18]; age 50 to 75 years on the date of invitation to participate; availability of GOHT platforms in the community; ability to communicate and to understand the study.

#### Exclusion criteria

Participants will be excluded if they have any of the following conditions: OA with severe joint deformity which needs surgery; other joint diseases, including rheumatoid arthritis and joint trauma; autoimmune diseases; chronic digestive disorders including chronic gastritis and chronic hepatitis; stroke; chronic kidney diseases; cancers; dementia or substantial cognitive impairment; psychotic illness; inability to give informed consent; unable to communicate or to understand the study; and no access to a GOHT platform.

### Recruitment

First, we will set up a research team, including rheumatologists, orthopedists, clinical researchers and nurses. Staff in the team are required to learn the details of the trial well through workshops and group discussions. In order to recruit participants, an invitation letter with a brief introduction of the trial will be sent to outpatients with knee OA. Advertising posters about the trial will be placed in the local newspapers, homepage of the hospital official website and WeChat (a popular social media application in China) official account. An overview of the trial and various announcements will also be available on these platforms. In the clinic, clinicians will be responsible for identifying the potential participants from the outpatients’ reception room, and will further make an overall assessment to decide whether they meet the requirements. Participants who meet the eligibility criteria will be recruited from February 2018 to June 2018.

### Sample size

This pilot study will be conducted without performing a formal sample size calculation, as the primary outcome is the feasibility of GOHT in the enhanced management for patients with OA. We plan to recruit 20 participants for each group, allowing for a 20% dropout over the period. As the recommended sample size for a feasibility pilot trial is 12 per group [19], we anticipate that the sample size of 40 participants will provide us with sufficient information about the feasibility of the intervention including the enrollment rate and the acceptability of the intervention.

### Randomization and blinding

Recruited participants will be randomly allocated to either the CT-GOHT group (intervention) or the CT group (control) at a 1:1 ratio. Random numbers will be generated using a computer software program run by an external statistician. Each of the random numbers with the group assignment will be written on a piece of paper and packed in a sealed envelope. After baseline assessment, participants will be provided an envelope according to the randomization sequence. The outcome assessors will be blinded to the participant randomization assignment.

### Intervention group (CT-GOHT group)

In addition to conventional therapy (celecoxib, 0.2 g daily), participants in the CT-GOHT group will receive a brief repeated GOH-based management every month via the GOHT platform. The web-based management is expected to last 20–30 min for an independent intervention, containing three broad segments: encouragement, educational lectures and medical issues. The clinicians who perform the intervention have received standardized training and should be familiar with the process and the detailed contents of an intervention. To ensure standardized interventions for each participant, standard operating procedures will be provided for the researches.

Participants will be required to attend follow-up clinics at 2 weeks, 3 months and 6 months after the initiation of the trial. When the symptoms get worse, participants will be allowed to get further treatment at any time.

#### Encouragement

This session aims to comfort patients and help them to face illness with great courage. The participants will be allowed to express their negative feelings related to OA. The clinician will encourage them to be optimistic and, to make them feel that they are being cared for and listened to.

#### Educational lectures

In this session, the participants will receive background knowledge of OA, including pathogenesis, risk factors, clinical manifestations, pharmacological therapies and prognosis of OA. Besides, some feasible non-pharmacological therapies, like dietary control, weight loss and proper exercise, will be recommended to the participants based on their personal conditions. The importance of long-term disease management in the rehabilitation of OA will also be emphasized. If there are any questions related to knee OA or the study, the participants are expected to receive responses from the clinician via GOH.

#### Medications

The clinician will emphasize the importance of medications in disease remission and remind participants to take medications on time at each GOHT visit. Besides, the clinician will tell participants the potential side effects medications may cause. If possible severe adverse reactions emerge, the participants should withdraw from the trial and get further treatments.

### Control group

Participants in the CT group will receive conventional therapies (celecoxib, 0.2 g daily). They will not receive any network-enhanced management via GOH. Similarly, participants will be required to attend follow-up clinics at 2 weeks, 3 months, 6 months after the initiation of therapy. Participants will be allowed to receive further treatments when their symptoms get worse. Any additional treatments received during the follow-up period will be recorded.

Participants in both groups are free to withdraw from the study at any time, but the withdrawal rates and reasons will be recorded.

### Outcome assessments

The outcome assessments will be made at baseline, 2 weeks and 3 month later after the initiation of the study and at the end of 6 months. All measurements of participants will be performed by a researcher blinded to the group allocation.

#### Primary outcome measures

**Fig. 3:**
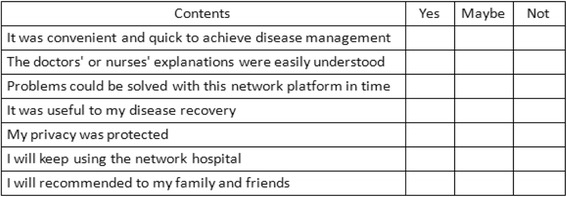
The satisfaction survey of Guangdong Online Hospital (GOH)

The primary outcome measurement is feasibility of the study. It will be evaluated by recording the proportion of patients with knee OA who meet eligibility criteria but decline to participate; the adherence to the intervention and the withdrawal rates. Besides, we will investigate the satisfaction in using GOHT among participants in the CT-GOHT group (Fig. 3).

#### Secondary outcome measures

The Western Ontario and McMaster Universities Osteoarthritis Index (WOMAC) [20] will be used to measure pain, stiffness and physical function in the knee. WOMAC, a self-administered health-status instrument for patients with knee OA, consists of 24 items within three subscales: pain (five items), stiffness (two items) and physical function (17 items). We will be use the VAS to measure the items of pain. The WOMAC scores will be standardized to a 0 (worst) to 100 (best) total score range.

The Multidimensional Fatigue Inventory (MFI) [21] will be used to evaluate fatigue. The MFI, widely used for measuring fatigue, is a 20-item self-report instrument. It covers the following dimensions: general fatigue, physical fatigue, reduced activity, reduced motivation and mental fatigue.

The Hospital Anxiety and Depression Scale (HADS) [22] will be used to assess anxiety and depression. The HADS is a widely used instrument to measure mental condition in many chronic diseases. It is a 14-item scale with seven items for anxiety (HADS-A) and seven for depression (HADS-D). Scores ranged from 0 to 21 for each subscale.

The Pittsburgh Sleep Quality Index (PSQI) [23] will be used to assess sleep quality. The PSQI, Global PSQI with a total score ranging from 0 to 21, is a standardized self-administered questionnaire for the assessment of sleep quality over the previous 1 month. It contains several sections to assess subjective sleep quality, sleep latency, sleep duration, sleep efficiency, sleep disturbances, use of sleeping medications and daytime dysfunctions.

### Ethics

This trial has been approved by the Human Research Ethics Committees of Guangdong Second Provincial General Hospital (2017-FS-032). This trial has been registered at the Chinese Clinical Trials Registry: ChiCTR1800014465. All study participants will be provided with written consents prior to participation. Written informed consent has been obtained from the patients for publication of this manuscript and accompanying images. All study-related information will be securely protected at the study site.

### Quality assurance and monitoring

**Fig. 4:**
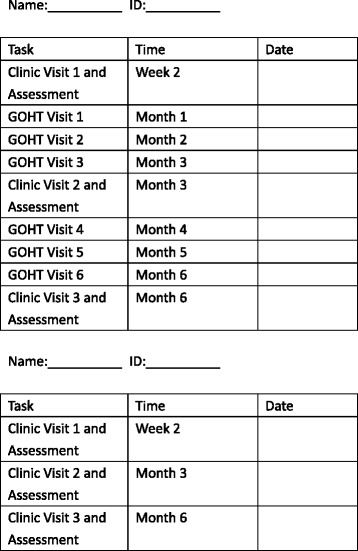
Task form for the conventional therapy plus a brief Guangdong Online Hospital intervention (CT-GOH) group (above) and the conventional therapy (CT) group (below)

To ensure that the study is of a high quality, all study procedures will be conducted in compliance with the protocol in the principles of the Good Clinical Practice (GCP) provided by the International Conference on Harmonization [24]. The researchers will learn to perform the study properly via a 2-day training workshop. They will also be given a written protocol and standardized intervention content documents. A detailed explanation of the study and a task form (Fig 4) will be provided to participants. Moreover, the outcome assessors will be blinded to group assignment.

**Fig. 5:**
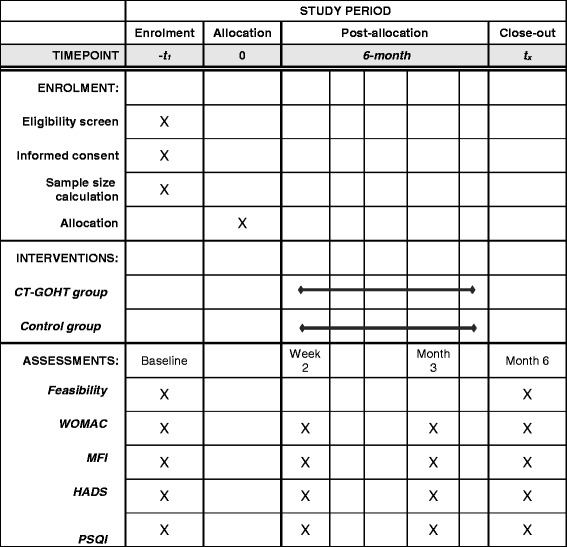
The schedule of enrollment, interventions, and assessments

The study progress and research data will be consistently monitored by an Independent Data Monitoring Board (IDMB), which will be independent from the study organizers. The IDMB will examine trial procedures, facilitate compliance with the study protocol, and ensure the data quality and safety.

### Withdrawal

Participants are free to withdraw from the study at any time, which will be informed during the consent process. If a participant chooses to withdraw, they will be asked to provide reasons and the reasons for withdrawal will be recorded. Besides, participants can discontinue the study at any time point if they experience any adverse events. The adverse events will be recorded.

### Planned statistical analysis

To evaluate the feasibility of the study, we will calculate the proportion of enrolled participants out of all eligible patients, participants who adhered to the intervention and the dropout rates. Besides, a patient satisfaction survey for the use of GOHT will be collected and analyzed.

Means and standard deviations will be used to describe between-group statistics of WOMAC, MFI, HADS and PSQI. All these variables will be analyzed using a repeated-measures mixed-effect model with terms for age, sex, Body Mass Index and treatments. The measurement data will be assessed for normal distribution using Shapiro-Wilk’s test for both groups. Independent samples’ *t* test will be used for the comparison of two-independent samples. Non-parametric tests (Mann-Whitney *U* test) will be used in cases of abnormal distribution. Statistical analysis will be processed by SPSS software (version 19.0). The categorical variables will be compared by a chi-square test. *P* < 0.05 will be accepted as the threshold for statistical significance.

## Discussion

Long-term disease management combined with both pharmacological and non-pharmacological therapies is essential for the treatment of chronic diseases. As a kind of long-term chronic disease management, telemedicine has shown effectiveness in the treatment of OA by providing multi-faceted interventions [25]. However, a considerable number of OA patients in China are unlikely to receive long-term treatment due to poor adherence, drug resistance or lack of disease knowledge. Furthermore, China is a large country with regional imbalance in economic development, which makes it difficult for patients from the remote areas to receive high-quality medical services. Recently, the Chinese Government has put forward a series of policies including the “Internet Plus” action plan to deal with this situation [26, 27].

This study is an exploratory trial in response to the Internet Plus action plan by providing disease management via GOHT. It will reveal the willingness of patients to participate in the forthcoming RCT, adherence to the interventions and the possible dropout rates. In addition, this study will collect preliminary information regarding the effects of GOHT in relieving knee pain, stiffness and physical dysfunction, in improving fatigue, anxiety, depression and sleep quality in patients with knee OA. These will be useful for power calculations in future larger-scale RCTs. The potential limitation is that the participants may not turn up on time for clinic visits and GOHT visits during this 6-month study. Therefore, every participant will be provided with a task form with the accurate time of their appointments stated. The schedule of enrollment, interventions, and assessments is explained in Fig. 5.

To the best of our knowledge, this is the first trial to explore the role of telemedicine in the management of OA patients in China. The findings from this study will contribute to better understanding of the practice of telemedicine in China. As a part of the Internet Plus action plan, our study has been encouraged by the provincial government and GOH is likely to provide telemedicine services for patients with other chronic diseases and help them with their health problems via a web-based disease management program. If GOH is feasible and effective in disease-enhanced management for patients with knee OA, a further larger-scale clinical trial will be conducted to test the effect of GOHT telemedicine on OA. This will eventually be beneficial for OA patients, especially for those in remote areas.

### Trial status

The trial is still pending at the time of manuscript submission.

## Additional file

Additional file 1: Standard Protocol Items: Recommendations for Interventional Trials (SPIRIT) 2013 Checklist: recommended items to address in a clinical trial protocol and related documents*. (DOC 120 kb)

## Abbreviations

ACR: American College of Rheumatology
CT: Conventional therapy
CT-GOHT: Conventional therapy plus a brief GOH-based telemedicine intervention
GCP: Good Clinical Practice
GOH: Guangdong Online Hospital
GOHT: Guangdong Online Hospital telemedicine
HADS: the Hospital Anxiety and Depression Scale
MFI: the Multidimensional Fatigue Inventory
OA: Osteoarthritis
PSQI: the Pittsburgh Sleep Quality Index
RCT: Randomized controlled trial
VAS: Visual Analogue Scale
WOMAC: Western Ontario and McMaster Universities Osteoarthritis Index
